# Digital mental health interventions for treating mental disorders in young people based in low-and middle-income countries: A systematic review of the literature

**DOI:** 10.1017/gmh.2024.71

**Published:** 2024-08-27

**Authors:** Janagan Alagarajah, Diana Ceccolini, Sydney Butler

**Affiliations:** 1 Faculty of Public Health and Policy, London School of Hygiene and Tropical Medicine, London, UK; 2 Centre for Global Mental Health, London School of Hygiene and Tropical Medicine, London, UK; 3 Independent Scholar

**Keywords:** young people, digital mental health interventions, low- and middle-income countries, systematic review, global mental health

## Abstract

Young people (YP) (between 10 and 24 years) are disproportionally vulnerable to developing and being affected by mental health conditions due to physical, social and emotional risk factors. YP in low-and middle-income countries (LMICs) have poorer access to, and quality of, mental health services compared to those in high-income countries. Digital mental health interventions (DMHIs) have been proposed as tools to address this burden of disease and reduce the global treatment gap in youth mental health outcomes. This study aimed to examine the evidence for DMHIs for treating mental disorders in YP based in LMICs. To do this, the author searched academic databases (MEDLINE, PsycINFO, Embase and Web of Science) for primary studies on DMHIs targeting YP in LMICs. Preferred Reporting Items for Systematic Reviews and Meta-Analyses criteria were followed. The quality of the studies was assessed using the Critical Appraisal Skills Programme) framework. A narrative synthesis methodology was used to summarise and explain the findings. The authors identified 287 studies of which 7 were eligible in the final review. The authors found evidence of the effectiveness of multiple forms of DMHI (especially internet-based cognitive behavioural therapy) on anxiety and depression outcomes. Studies reported a lack of long-term benefits of treatment, high dropout rates, and did not include key geographical settings or data on cost-effectiveness. No studies were judged to be of high quality. This review highlights the available evidence showing that DMHIs can improve mental health outcomes for YP in LMICs, but due to the limited number of studies and lack of high-quality data, increased adoption and scaling up of digital interventions require more rigorous studies showing clinical effectiveness and ability to provide return on investment.

## Graphical abstract

**Figure:**
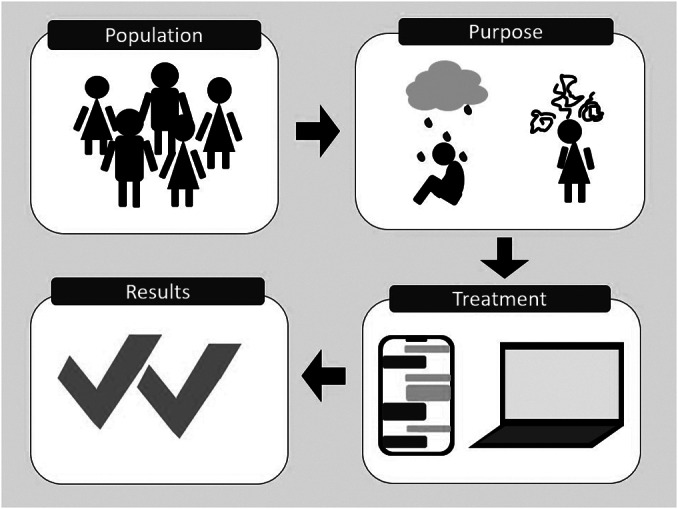

## Impact statement

Young people have an increased vulnerability to mental health conditions, and those living in low- and middle-income countries face disproportionate barriers in accessing high quality mental health care. Given increasing digital connectivity in the global south, digital mental health interventions (DMHIs) show promise in improving mental health outcomes for these populations by circumventing key barriers to care. In this systematic review, we evaluate the quality and availability of evidence on the effectiveness of DMHIs for young people and use this to provide evidence-based policy recommendations to improve youth mental health outcomes. Our findings show evidence of the effectiveness of multiple forms of DMHI (especially internet-based cognitive behavioural therapy) on anxiety and depression outcomes. At the same time, our results show a lack of high-quality studies on the topic, characterised by high dropout rates, small sample sizes and insufficient data on the statistical significance of treatment effects and long-term benefits of treatment. Our findings highlight that DMHIs have the potential to improve youth mental health outcomes in these settings but given the lack of robust data, increased adoption of these technologies would require further research on the topic.

## Introduction

Young people (YP) make up around a quarter (1.8 billion) of the world’s population, with almost 90% living in low- and middle-income countries (LMICs), where they constitute up to 50% of the population (UNFPA, 2014). YP, defined as those aged 10–24 by the World Health Organisation (WHO), are disproportionately affected by mental health issues (WHO, 2024). Around 50% of mental health conditions start by age 14, and 75% by age 24, and around 1 in 5 adolescents experience a mental health condition each year (Kessler et al., 2005), resulting in over 250 million YP globally having a mental health disorder (IHME, 2023). The Covid-19 pandemic and associated lockdowns have further exacerbated this burden (Racine et al., 2021).

YP are especially vulnerable to mental health problems due to exposure to physical, emotional and social risk factors, such as pressure from peers to conform, exploration of identity, stigma, discrimination, lack of access to quality mental health services, poverty, abuse and violence (Patel et al., 2007; WHO, 2020). Unfortunately, most mental illnesses among YP remain undiagnosed and untreated due to barriers to accessing and seeking care (Lehtimaki et al., 2021; UNICEF, 2021). YP in LMICs are disproportionately affected by this burden, due to fragmented and lower-resourced healthcare systems, poverty, stigma, lack of government policy, inadequate funding and a paucity of trained clinicians (Kieling et al., 2011; Rathod et al., 2017; Wainberg et al., 2017). The mental health treatment gap, defined as the difference between the number of people who need care and those who receive it (Jansen et al., 2015), is particularly significant for YP in LMICs, reaching rates of up to 90% (The WHO World Mental Health Survey Consortium, 2004; Duarte et al., 2022).

Digital mental health interventions (DMHIs), defined as ‘information, support and therapy for mental health conditions delivered through an electronic medium with the aim of treating, alleviating or managing (mental health) symptoms’ (Torous et al., 2021), are a viable alternative to face-to-face mental healthcare. These interventions can be delivered via multiple platforms, such as smartphone apps, online programmes, text messaging, telepsychiatry and wearable devices such as smart watches (Carter et al., 2021). Although YP living in LMICs have limited access to mental healthcare, many have access to digital technologies (WHO, 2020), at increasingly younger ages (Kardefelt Winther et al., 2019). Given that wireless connectivity in LMICs is becoming more widely available (The World Bank, 2024), and that smartphones are becoming cheaper, people in LMICs are increasingly able to access the internet (Kemp, 2020), making DMHIs a feasible solution to this treatment gap.

Effective DMHIs have the potential to help address the global inequality in the provision of mental health services, providing greater accessibility, acceptability, affordability, confidentiality and flexibility, leading to improved access to care (Wallin et al., 2016). By meeting the WHO criteria for YP-friendly interventions, namely availability, accessibility, equitability (e.g., non-judgmental care), acceptability (e.g., provision of confidential and youth-centred care) and appropriateness (Mazur et al., 2018), DMHIs can improve YP’s empowerment, participation and help-seeking behaviours (Shortliffe, 2016). Additionally, they could counter mental health stigma and provide safe and confidential care in cases where YP may fear social isolation or other inhumane responses to their mental illness (Semrau et al., 2015).

Despite their potential, there is limited research on DMHIs in LMICs, potentially due to researchers and clinicians prioritising clinical care over research output in resource-scarce healthcare systems (Kar et al., 2020; Lehtimaki et al., 2021). Additionally, there is a lack of governance and regulation over the use of DMHIs to improve YP’s mental health in LMICs (Petersen et al., 2017). These barriers may prevent the development, implementation and evaluation of such interventions in LMICs.

Until recently, DMHIs have mainly been developed for and used in high-income countries (HICs), where they have been found to be effective at reducing symptoms of mental health conditions such as depression (Firth et al., 2017), psychosis (Gire et al., 2017) and other severe mental illnesses (Naslund et al., 2015), while also improving medication adherence (Rootes-Murdy et al., 2018). Evidence of their effectiveness in LMICs is scarce (Larsen et al., 2019), limiting their applicability in these settings (Henrich et al., 2010). To understand opportunities for DMHIs for YP in LMICs, it is therefore essential to examine studies from these settings (Carter et al., 2021), given the under-prioritisation of mental health research (Becker and Kleinman, 2013) and the lack of governance and regulation around DMHIs (Petersen et al., 2017).

## Aims and objectives

To respond to the opportunities offered by DMHIs for YP in LMICs, comprehensive identification and assessment of the available evidence base is required. However, no literature reviews were found investigating this topic. Therefore, the overall aim of this review is to examine the evidence for DMHIs for treating mental disorders in YP in LMICs.

The specific objectives of the review are to:

1. Evaluate the clinical effectiveness of DMHIs on mental health symptoms for YP in LMICs.
2. Assess the availability and quality of the current evidence on DMHIs focusing on YP’s mental health outcomes based in LMICs.
3. Provide practice and research recommendations for the use of DMHIs focusing on YP’s mental health outcomes based in LMICs.

## Methods

Preferred Reporting Items for Systematic Reviews and Meta-Analyses (PRISMA) reporting criteria were followed (Page et al., 2021).

### Eligibility criteria

Eligibility criteria for this study (Table 1) were based on a modified version of the Population, Intervention, Control, Outcome framework (CRD, 2009; Methley et al., 2014).

**Table 1.** Eligibility criteria for studies

	Inclusion criteria	Exclusion criteria
Population	Average age of participants between 10 and 24 years (as per WHO definition of YP; WHO, 2024) Participants diagnosed with specific mental health conditions (as per ICD–11 criteria; WHO, 2023) and/or participants reporting generalised mental health outcomes (e.g., psychological distress, functioning/functional disability, quality of life and locally defined mental health outcomes) Mental health conditions are the primary disorders in the study	Average age <10 or >24 years. (Studies were also excluded if they included data from YP that were not disaggregated with data from other age groups.) Studies focus on the parents/carers of YP with mental health problems Mental health conditions are the secondary disorders in the study
Intervention	DMHIs defined as ‘information, support, and therapy for mental health conditions delivered through an electronic medium with the aim of treating, alleviating or managing (mental health) symptoms’ (Torous et al., 2021) All study types including randomised controlled trials, pilot trials, case control studies and naturalistic studies Primary data DMHI is the main component of intervention Interventions aim at treating YP with mental health conditions	Trial protocols, opinion pieces, case studies, qualitative content analysis, clinical guidelines and literature reviews Secondary data Digital intervention is not the main component of the intervention Intervention is not digitally based Interventions aim at screening/preventing mental health conditions rather than providing treatment
Control	Active control (e.g., non–digital intervention) or passive control (e.g., placebo/waitlist control/no treatment)	
Outcome	Clinical effectiveness of mental health interventions (measured using validated scales, e.g., depression scales such as PHQ–9, or anxiety scales such as GAD–7)	Other outcome measures e.g., feasibility, acceptability
Setting	LMICs (as per World Bank criteria for 2023; The World Bank, 2023)	HICs (as per World Bank Criteria for 2023; The World Bank, 2023)
Publication	Published in academic journals English language No time limits were applied	Grey literature Non–English literature

Abbreviations: DMHI, Digital Mental Health Intervention; GAD-7, general anxiety disorder-7; HICs, high income countries; ICD-11, international classification of diseases 11th revision; LMIC, low- and middle-income country; PHQ-9, patient health questionnaire-9; YP, young people.

### Search strategy and selection criteria

**Figure 1.**
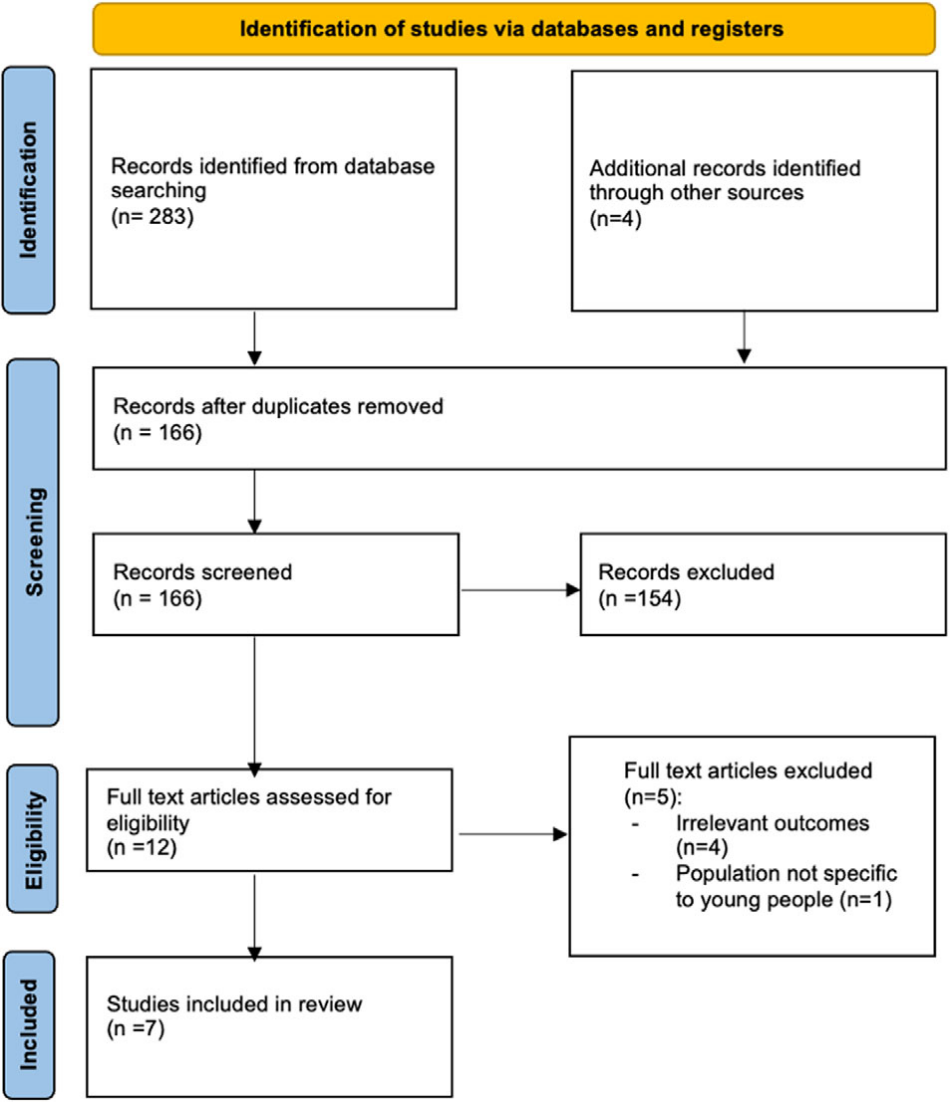
PRISMA flowchart.

The review was conducted using a predefined protocol based on the PRISMA reporting criteria (Page et al., 2021), with key stages being identification, screening, assessing eligibility and inclusion of studies (Figure 1). JA conducted an electronic review of the literature from the MEDLINE, Embase, Web of Science and PsycINFO databases, based on recommendations from the London School of Hygiene & Tropical Medicine (LSHTM) library staff (Table 2). DC re-ran all the searches as the second reviewer to minimise bias. JA and DC also hand-searched reference lists of all identified full text studies to manually identify relevant publications.

**Table 2.** Number of articles found

Name of journal	Number of articles found
Medline	99
Psychinfo	53
Embase	114
Web of Science	17

The authors used a combination of keywords such as (“digital,” “mHealth,” “eHealth,” “web-based,” “internet-based,” “mobile phone,” “text message,” “SMS,” “artificial intelligence”) AND (“adolescen*,” “youth” “young,” “child,” “student”) AND (“mental health,” “wellbeing”). An LMIC filter was used to select relevant studies. For a full list of search terms, please see Supplementary Material S1.

Identified references were screened by JA by conducting an abstract and title search based upon the eligibility criteria (Table 1). Full texts were assessed for final inclusion by JA. This process was repeated by the second reviewer (DC), reaching the same conclusions.

### Data extraction

JA extracted data from the studies, using a data extraction form (Table 3). Data were collected on the study context; population group; outcome(s) of interest; methods (sample size, study design, intervention type, control group, theoretical approach); targets (inclusion/exclusion criteria, participant characteristics); intervention (mental health issues addressed, technological approaches used, study setting, number of sessions, content, presence of mental health support) and impacts (evaluation methods, primary/secondary outcome measures and key findings).

**Table 3.** Included studies

Author	Country	Sample size (*n*), study design, intervention type	Control group	Theoretical basis	Participant characteristics	Mental health outcome(s)	Platform	Setting	Frequency/duration of intervention	Content	Evaluation methods	Primary and (relevant) secondary outcome measures	Key findings (clinical effectiveness)
Moeini et al. (2019)	Iran	n = 128 RCT Depression improvement programme (guided)	Not specified	Social Cognitive Theory/CBT	Mean age in the intervention and control groups were 16.2 and 16.5, respectively	Depression	Internet–based intervention	Schools	Eight 30–min sessions over 6 months.	1) Modules on: awareness–raising, positive psychology, problem–solving, thoughts and feelings, relaxation, physical exercise and lifestyle modifications 2) Delivered via videos, animations and PowerPoint slides	ITT	Primary outcome: Depressive symptoms (measured usingCES–D tool)	DMHI group reported a statistically significant (p < 0.05) improvement on the CES–D score at baseline (Mean = 22.6, SD = 10.9) to 12 weeks (Mean = 18.5, SD = 14.0). However, these results seem to have attenuated by 24 weeks (Mean = 19.5, SD = 10.9)
Newman et al. (2021)	India	n = 222 RCT Guided, Internet–based self–help intervention for GAD symptoms (’Lantern’)	Waitlist	CBT	Mean age was 19.90 years, 153 males (68.9%), 68 females (30.8%), and one trans female	GAD, Depression	Internet–enabled computer, mobile phone or tablet	University	3 month long intervention consisting of 40 10–min sessions)	Modules on: introduction to anxiety, automatic thoughts, cognitive reframing, introduction to behaviour change, imaginal exposure, situational exposure, mindfulness, and habit formation	ITT	Primary outcome measure: GAD symptom severity (measured usingGAD–Q–IV) Secondary outcome measures: 1) Worry (measured using PSWQ) 2) Depressive symptoms (measured using DASS depression subscale)	1) DHMI group experienced significant reductions on the GAD–Q–IV (ß = −3.27, SE = .31, Z = −10.44, p ˂< .001, d = −1.96), PSWQ (ß = −7.66, SE = 1.73, Z = −4.43, p < .001, d = −.62), and DASS depression (ß = −3.65, SE = .70, Z = −5.24, p < .001, d = −.75) 2) Participants in the control group experienced a statistically significant but smaller reduction on the GAD–Q–IV (ß = −1.94, SE = .33, Z = 5.91, p < .001, d = −.84) and did not experience significant reductions on the PSWQ (ß = .37, SE = 1.91, Z = .20, p = .841, d = .03) or DASS Depression (ß = .25, SE = .79, Z = .32, p = .753, d = .04)
Ofoegbu et al. (2020)	Nigeria	n = 192 RCT Guided internet–assisted intervention (GIAI)	Usual care	CBT	Average age in treatment group 24.21 and non–treatment group 23.78	Depression	Internet–based	University	10 week intervention	Self–guided (videos, audios, and print materials for depression treatment) with support from therapists (twice a week) Sessions focussed on psychoeducation, interactive peer support, cognitive disputation, behavioural homework assignments, roleplay, and depression management	ANOVA	Primary outcome measure: Depressive symptoms (measured using BDI–II scale)	1) Significant reduction in depressive symptoms among the participants in the treatment group when compared to their counterparts in the usual–care control group, F (1111) = 254.56, p < .001, h2p ¼: 956 2) At follow–up (4 weeks post intervention) there was a significant reduction in depressive symptoms among participants in the treatment group compared to those in the usual–care control group, F (1111) = 261.89, p < .001, h2p ¼: 960
Osborn et al. (2020)	Kenya	n = 103 RCT Digital single session intervention (‘Shamiri’) (guided)	Study–skills control intervention	Not stated	Not stated	Depression, anxiety	Internet–based intervention	High school	One session	Mindset, gratitude, and value affirmation exercises	ITT	Primary outcome measures: 1) Adolescent depressive symptoms (measured using PHQ–8 scores), 2) Adolescent anxiety symptoms (measured using GAD–7 scores) 2) Adolescent mental well–being (measured using WEMWBS 3) Happiness and Optimism (measured using EPOCH scale) Secondary outcome measures: 1) Depressive symptoms for the subsample with elevated depressive symptoms at baseline (PHQ–9) 2) Anxiety symptoms for the subsample with elevated anxiety symptoms at baseline (GAD–7)	1) Compared to the control group, participants in the DMHI group experienced greater reduction in adolescent depression symptoms in both the full sample (p = 0.028, d = 0.50) and a sub–sample of youths with moderate–to–severe depression symptoms (p = 0.01, d = 0.83) from baseline to 2–week follow–up 2) The DMHI had no significant effects on anxiety symptoms, well–being or happiness
Salamanca–Sanabria et al. (2020)	Colombia	n = 214 RCT Culturally adapted cognitive behavioural internet–delivered treatment (guided)	Waitlist control	CBT	Total average age 22.15	Depression, anxiety	Internet–based intervention	College	3 months of iCBT	Seven modules of CBT self–monitoring, behavioural activation, cognitive restructuring, and challenging core beliefs	ITT	Primary outcome measure: Depression (as measured by the PHQ–9) Secondary outcome measure: Anxiety (as measured by the GAD–7 questionnaire)	1) PHQ–9: those in the treatment group showed statistically significant reductions in depressive symptom scores (p < 0.001) following treatment that were maintained at 3 month follow–up 2) GAD–7: significant differences in the GAD–7 score change recorded from baseline to posttreatment between the groups (p ≤ .03) in favour of the treatment group
Sun et al. (2022)	China	n = 114 RCT Mindfulness–based mobile health intervention (guided)	Time– and attention matched social support–based mHealth control	MBI	Mean age 22.21 years old. Majority female	Depression, anxiety	Internet–based delivery using apps (Zoom and WeChat)	University	Four weekly, 1 hour long sessions	Experiential and group learning of mindfulness, didactic learning about mindfulness and audio–based daily practice	ITT	Primary outcomes: 1) Anxiety (measured using GAD–7) 2) Depression (measured using PHQ–9)	1) In terms of anxiety, a greater reduction was found in the intervention group from baseline to follow–up (proportion reduced from 63.2% to 9.6%), which was greater than the control group (57.9% to 27.7%). The difference between groups was statistically significant (p = .020) 2) Reduction of depressive symptoms in intervention group from baseline to follow–up (73.7% to 17.3%) compared to the control group (71.9% to 34.0%) was not statistically significant (p = .056)
Wannachaiyakul et al. (2017)	Thailand	n = 84 RCT Computerised CBT programme for reducing depression among YPs with delinquency problems (guided)	Usual activity control group	CBT	The mean age in the experimental and control groups were both 17.74 years. Most of participants were male; finished junior high school; in confinement for the first time; and involved in drug cases	Depression	Computerised platform	Youth detention centre	One session per week lasting 45–60 min for 6 weeks	Tasks focusing on learning about depression, mood monitoring and developing emotional literacy using case studies, interactive exercises and self–reflection	ANOVA, T test (did not specify if ITT/per protocol methods were used)	Primary outcome: Symptoms of depression (as measured by the PHQ–9)	Participants in the intervention group after entering the program, and 1 and 2 months after the intervention had significantly (p < 0.05) lower mean scores of depression than before receiving the programme Additionally, those in the intervention group had a significantly lower mean score of depression than that of the control group immediately after completing the programme (p < 0.05)

Abbreviations: ANOVA, analysis of variance; BDI-II, Beck’s Depression Inventory; CBT, cognitive behavioural therapy; CES-D, centre for epidemiologic studies depression scale; DASS, depression anxiety and stress scale; DMHI, Digital Mental Health Intervention; GAD, generalised anxiety disorder; GAD-7, general anxiety disorder-7; GAD-Q-IV, generalised anxiety disorder questionnaire IV; HICs, high income countries; iCBT, internet-based cognitive behavioural therapy; ICD-11, international classification of diseases 11th revision; ITT, intention-to-treat analysis; LMIC, low- and middle-income country; MBI, mindfulness based intervention; mHealth, mobile health; PHQ-8, patient health questionnaire-8; PHQ-9, patient health questionnaire-9; PSWQ, Penn State Worry Questionnaire; RCT, randomised control trial; WEMWBS, Warwick-Edinburgh Mental Wellbeing Scale; YP, young people.

As only randomised control trials (RCTs) were identified, JA used Critical Appraisal Skills Programme’s (CASP’s) RCT criteria as a validated quality assessment framework to appraise the quality of identified studies (see Supplementary Material S2) (CASP, 2020). CASP was selected over other assessment tools as it focuses on study validity, results and clinical relevance, which align with the review’s objectives (CASP, 2020). We utilised Vogel’s (2013) criteria to evaluate the quality of studies, categorising them as high, medium or low quality. Although we initially planned to exclude any study identified as “low quality,” none met this criterion upon evaluation. Consequently, all studies were included in the analysis.

### Data synthesis

A descriptive analysis was conducted, based on the study objectives. Due to the expected heterogeneity of the included interventions, outcome types, measures and study designs, a quantitative synthesis (meta-analysis) of the findings was not deemed appropriate. JA therefore synthesised evidence from the articles describing the clinical effectiveness of DMHIs using a narrative synthesis approach.

## Results

### Selection of included studies

The initial search yielded 283 results. After excluding duplicate references, the number of articles was reduced to 166. The manual search yielded an additional four articles for eligibility assessment. A total of seven articles were finally included (Wannachaiyakul et al., 2017; Moeini et al., 2019; Ofoegbu et al., 2020; Osborn et al., 2020; Salamanca-Sanabria et al., 2020; Newman et al., 2021; Sun et al., 2022) (see Figure 1 for PRISMA flowchart [Page et al., 2021]).

### Characteristics of included studies

Details of the final seven eligible studies are provided in Table 3. The studies were all conducted between the years 2017 and 2022 in five geographic regions (Africa n = 2, Southeast Asia n = 2, South Asia n = 1, South America n = 1, Middle East n = 1). The mean age of participants varied from 16.2 (Moeini et al., 2019) to 24.21 years (Ofoegbu et al., 2020). Several studies were based in universities (Ofoegbu et al., 2020; Salamanca-Sanabria et al., 2020; Newman et al., 2021; Sun et al., 2022); however, other settings such as schools (Moeini et al., 2019), high schools (Osborn et al., 2020) and a youth detention centre (Wannachaiyakul et al., 2017) were also studied. All studies used a RCT design. Three studies were specifically focused on depression, and four studies on depression and anxiety. Notably, no studies were found evaluating DMHIs focussed on any other psychopathology. All but one study only included participants with mild–moderate symptoms, excluding those with severe symptoms or comorbidities.

Studies used different theoretical concepts to underpin interventions, such as mindfulness (n = 1), cognitive behavioural therapy (CBT; n = 5) and social cognitive theory (n = 1) All reviewed interventions were accessible from mobile devices or computers and used internet-based platforms, except for a computerised platform evaluated by Wannachaiyakul et al. (2017). All identified interventions also involved either new content and/or adaptations of existing evidence-based psychosocial treatments. For example, Salamanca-Sanabria et al. (2020) culturally adapted an existing programme to create a Colombian version of internet-based CBT (iCBT), while Sun et al. (2022) used a popular Chinese social media platform (WeChat) to deliver a mindfulness intervention. Digital interventions included a range of content (e.g., challenging core beliefs, increasing knowledge about mental health, value affirmation exercises) using a range of multimedia options (e.g., videos, animations, presentations). All interventions were externally guided or supported. The interventions lasted between a single session (Osborn et al., 2020) and 6 months (Moeini et al., 2019). Dropout rates in the intervention group ranged from 9% (Sun et al., 2022) to 91% (Salamanca-Sanabria et al., 2020). Two studies (Wannachaiyakul et al., 2017; Osborn et al., 2020) had no loss to follow-up. No studies reported on the cost-effectiveness or design elements of DMHIs.

Studies were found to have selection bias through loss to follow-up (e.g., Moeini et al., 2019 reported a 30% drop out rate in the intervention group), and recruitment via self-selection (e.g., Osborn et al., 2020 recruited all students who were interested in the study). Only three studies (Wannachaiyakul et al., 2017; Moeini et al., 2019; Newman et al., 2021) reported sample size calculations, and six studies (Wannachaiyakul et al., 2017; Moeini et al., 2019; Osborn et al., 2020; Salamanca-Sanabria et al., 2020; Newman et al., 2021; Sun et al., 2022) had small sample sizes that may have led to underpowered results. Moreover, only four studies (Ofoegbu et al., 2020; Osborn et al., 2020; Salamanca-Sanabria et al., 2020; Sun et al., 2022) reported precision estimates. There may also have been an element of placebo or Hawthorn effect in some studies. For example, those in the (waitlist) control group in the Newman et al. (2021) study also experienced a statistically significant reduction in their anxiety scores.

### Effectiveness of DMHIs for depression and anxiety

Three studies focussed specifically on depression. Ofoegbu et al. (2020) evaluated a 10-week long internet-based intervention with Nigerian university students using CBT principles. They found significant reductions in depression scores (p < .001), which were maintained at 4-week follow-up (p < .001). Moeini et al. (2019) administered a web-based intervention to school children underpinned by social cognitive theory/CBT principles in Iran over 6 months. Statistically significant improvement in depressive symptoms between baseline and 12 weeks were found (p < .05). This improvement did not continue past 24 weeks. Wannachaiyakul et al. (2017) utilised a 6-week long computerised intervention with inmates at a youth detention centre in Thailand. They found that depression scores reduced after entering the programme, and at 1- and 2-month follow-up (p < .05).

Four studies addressed both anxiety and depression. Newman et al. (2021) evaluated a CBT-informed intervention for Indian university students with generalised anxiety disorder over 3 months. The intervention was associated with statistically significant reductions in anxiety (p ˂ .001) and depressive symptoms (p ˂ .001). Sun et al. (2022) administered a mindfulness-based digital intervention using apps to Chinese university students with depression and anxiety symptoms over 4 weeks. This digital intervention led to statistically significant reductions in anxiety (p < .05), but not in depressive symptoms. Salamanca-Sanabria et al. (2020) implemented a 3-month long CBT-based digital intervention among Colombian university students with depression. They found that treatment with iCBT led to significant reductions in depression (p < .001) and anxiety (p < .05) symptoms. Osborn et al. (2020) utilised a single session internet-based intervention on adolescents in a Kenyan high school. The intervention produced a statistically significant reduction in depressive symptoms from baseline to 2 week follow-up (p < .05), but not in anxiety symptoms. This was the only study to include those with moderate to severe depressive symptoms. Given the heterogeneity of included studies, comparing efficacy among interventions was not possible.

### Quality assessment of included studies

The author assessed studies based on the CASP criteria (see Appendix 2) (CASP, 2020). All seven studies were judged to be of moderate quality. Aspects of the CASP criteria that studies performed well in were clearly addressing a focused research question (n = 6); detailing the method of randomisation (n = 7); accounting for loss to follow-up (n = 5); ensuring that both intervention and control groups were treated equally apart from the intervention (n = 7); ensuring comprehensive reporting of intervention effects (n = 7) and ensuring that the benefits of the trial outweighed the harms/costs (n = 7). However, areas of weakness included a lack of blinding of participants (n = 3); a lack of reporting around similarity between groups at the start of the trial (n = 4) and a lack of reporting on the precision of the treatment effect (n = 4).

## Discussion

The present systematic review aimed to evaluate the clinical effectiveness of DMHIs on the mental health symptoms of YP in LMICs, assess the availability and quality of the current body of evidence on the topic, and provide practice and research recommendations for the use of DMHIs for YP in LMICs. With regard to the effectiveness of DMHIs, all studies included in this review reported statistically significant improvements in YP’s mental health outcomes. The use of the ‘gold standard’ RCT methodology in all identified studies supports confidence in their results. Notably, no studies were found reporting a worsening of symptoms, negative acceptability or dissatisfaction with DMHIs. However, this lack of negative findings may reflect publication bias favouring positive results. Future reviews could use a funnel chart to evaluate this. Regardless, we must apply caution when drawing conclusions from these studies, given the limitations of the studies reviewed.

No DMHIs identified in the review targeted other types of psychopathology aside from depression and anxiety. This is consistent with findings from a literature review focussing on DMHIs for adults in LMICs (Carter et al., 2021). All but one study excluded those with severe symptoms, comorbidities, and those on psychotropic medication, psychological treatment or displaying self-harm/suicidal ideation. These factors limit the generalisability of the findings in three ways. Firstly, symptoms that were excluded from studies such as suicidal ideation are common in YP with depression/anxiety (Avenevoli et al., 2015). By excluding these participants, study findings could only apply to a small subset of patients. Secondly, comorbid mental health conditions are common in YP (Angold and Costello, 1993), further limiting the target population for these studies. Thirdly, the study findings are not applicable to a significant proportion of YP with more severe mental health issues (Tsehay et al., 2020). The studies in this review also largely targeted university students, making it difficult to draw conclusions about the effectiveness of DMHIs for children and adolescents. The heterogeneity in intervention types, outcome measures and study durations limited the possibility of conducting a meta-analysis, which could have strengthened conclusions about DMHIs’ effectiveness.

Considering the high recurrence rates and chronicity of common mental disorders, it is also vital to understand whether DMHIs have long-term effects (Koopmans et al., 2011). This review found that DMHIs were not always able to sustain improvements in mental health symptoms. Moreover, the lack of meaningful long-term follow-up periods found in this review (mostly under 6 months), similar to the findings from a review of studies on DMHIs in HICs (Lehtimaki et al., 2021), does not allow for a valid assessment of sustained treatment effects (Clarke et al., 2015). Despite the paucity of long-term data, a meta-analysis of HIC studies found three DMHIs showing significant improvements in depressive symptoms in YP after 6 months (Välimäki et al., 2017). However, the quality of data from HICs may be worse than that from LMICs. HIC studies were judged to have ‘consistently low quality’ in a large systematic overview (Lehtimaki et al., 2021), while no studies were judged to be of low quality in this review. Furthermore, a systematic review (Grist et al., 2017) identified key limitations in HIC studies that were similar to those found in this review, such as small sample sizes, limited participant blinding and recruitment via self-selection.

Although all studies included in this review reported statistically significant improvements in YP’s mental health outcomes, the current review found varying effect sizes. This may be due to variations in recruitment strategy (Harith et al., 2022), as web-based recruitment generally shows larger effect sizes than subject pool recruitment (Harrer et al., 2019). Sun et al. (2022) (reporting a large effect size) recruited online, while Moeini et al. (2019) (reporting a small effect size) recruited via a subject pool. Those recruited online may already be more interested in DMHIs and could engage better with interventions than those recruited from a subject pool, leading to larger effect sizes.

Variation in effect size may also be influenced by participant adherence, as higher rates of adherence are generally associated with better treatment outcomes (Conley et al., 2016). Participants who adhere to an intervention may receive an increased ‘dose’ of an intervention leading to improved outcomes compared to those that drop out. The small effect size in the Moeini et al. (2019) study might therefore be related to the high dropout rate (30%) in the intervention group. Comparably to this review’s findings, literature from HICs reported low adherence and high dropout rates (Lehtimaki et al., 2021). Completion rates in this review varied from 9% to 100%, similar to completion rates of 10%–94% found in a systematic review of DMHIs in HICs (Välimäki et al., 2017). Notably, the two studies that reported no loss to follow-up in our review either used a single session intervention (Osborn et al., 2020) or an incarcerated population that may have had limited choice regarding participation (Wannachaiyakul et al., 2017). Although HIC data also show that loss to follow-up could be lowered by using supported interventions, this review’s findings showed that supported interventions can still report high dropout rates (Clarke et al., 2015).

Although intervention design may impact the effectiveness of DMHIs (Chandrashekar, 2018), it is difficult to evaluate the effectiveness of specific styles of intervention design in this review as none of the studies reported on specific design elements used. iCBT has been found to be as effective or more in treating YP’s anxiety and depression than traditional CBT in HICs (Ebert et al., 2015; Podina et al., 2016). This review’s outcomes support these findings. However, contrary to this review, Lehtimaki et al. (2021) found that apart from iCBT, there was inconclusive evidence for other types of DMHIs (e.g., mobile apps) in treating YP’s mental health issues in HICs. This could be because other digital interventions are highly tailored to the population group, country, and setting, which might have hindered appropriate comparisons between interventions.

HIC literature also supports the review’s findings on the lack of published data on DMHIs’ cost-effectiveness (Lehtimaki et al., 2021). This could act as a barrier to implementing DMHIs in LMICs, as decision-makers may be reluctant to invest in an intervention when return on investment is unclear. Moreover, given financial constraints in LMICs, proving that an intervention is cost-effective could be key to its implementation.

### Recommendations for future research and practice in LMICs

This review confirms the clinical effectiveness of DMHIs for YP in low-resource settings. They are potentially cost-effective treatment options that could permit large-scale dissemination and reduce healthcare worker burden (De Kock et al., 2022). With most of the world’s social media users located in LMICs (Shewale, 2023), there is significant potential to use DMHIs to reach large numbers of YP and support mental health promotion efforts and service delivery in these settings (Naslund et al., 2020). However, despite the compelling evidence presented in this review, uptake and integration of DMHIs in health systems remains low, especially in LMICs (Torous et al., 2018). Moreover, framing DMHIs as innovative approaches may lead to inappropriate enthusiasm to develop and implement technological solutions over other forms of intervention (WHO, 2020), further exacerbating health inequalities.

As per WHO digital health system strengthening guidelines (WHO, 2019), careful evaluation of benefits and harms is vital to avoid negative impacts on LMICs. Digital interventions that are incompatible with the needs and preferences of YP in LMICs may lead to inappropriate resource use, reduced clinical efficacy, and exacerbation of health inequalities (WHO, 2019). Given the digital divide between HICs and LMICs, the implementation of DMHIs without being coupled with campaigns (e.g., the United Nations’ Sustainable Development Goal [SDG] 9.c: “strive to provide universal and affordable access to the Internet in least developed countries by 2020”; [UN, 2015; UNDP, 2017]) to increase internet access may also exacerbate inequalities in access to mental health care and outcomes (UNICEF, 2017; ITU, 2023). Despite increases in global internet access and mobile phone use, connectivity in low-resource contexts still remains behind that of high-income contexts and international targets set under the Connect 2020 Agenda (ITU, 2014; UNDP, 2017; GSMA, 2022).

There are also inequalities in internet access within LMICs. For example, in low resource contexts, women, rural residents, older adults, persons with disabilities and those from lower socio-economic groups have the lowest rates of internet access (Naslund et al., 2019; GSMA, 2021, 2022). There are also regional and sub-regional inequalities in internet access within LMICs. For instance, sub-Saharan Africa has the lowest internet connectivity globally, and within this region, central Africa specifically has the lowest mobile broadband coverage on the continent (GSMA, 2022). Disparities in internet access between HICs and LMICs in addition to those within LMICs may therefore act as a barrier to the uptake of these technologies by vulnerable populations in low resource settings.

Given the digital divide in low resource contexts, opportunities for effective implementation of DMHIs in these settings may be maximised by equitably allocating resources (e.g., electricity, connectivity, and data) to address disparities in internet connectivity (ITU, 2021, Public Health Insight, 2023). Governments should deliver targeted policies to increase the uptake of DMHIs in underserved groups (e.g., increasing women’s internet connectivity through increasing access to digital resources, financial support and digital literacy skills; UNCTAD, 2023). Governments should also strategically align mental health care priorities with existing SDGs related to increasing internet access (ITU, 2021; Public Health Insight, 2023). For example, maximising access to technology (outlined in SDG 9) could also increase access to evidence-based mental health services (SDG 3) (UN, 2015; ITU, 2021; van Kessel et al., 2022; ITU and UNDP, 2023; Public Health Insight, 2023). By highlighting the co-benefits of digital health technologies, it may improve funding, roll out and implementation of innovative DMHIs in LMICs.

DMHIs may also increase the burden on healthcare staff. In this review, all identified interventions involved some level of external support. Although associated with improved treatment efficacy, implementation of an intervention with external support may be inappropriate in resource-constrained LMIC contexts (Grist et al., 2019). Investment in DMHIs may also be associated with an opportunity cost, potentially leading to reductions in funding to other elements of already strained LMIC health systems (WHO, 2019). Finally, given the lack of data on the cost-effectiveness of DMHIs, it is difficult to assess the financial burden of DMHIs on LMIC health systems (Lehtimaki et al., 2021). A potential method of minimising costs and maximising benefits to LMIC healthcare systems could be to use trained non-specialist helpers to reduce resource use while providing digital support, which may increase the intervention’s efficacy and adherence (Hoeft et al., 2018). A DMHI called ‘Step-by-Step’ created by the WHO for adult Syrian refugees in Lebanon has already used this approach, leading to improvements in depressive symptoms (Cuijpers et al., 2022).

Although data show that some DMHIs are as effective as traditional mental health services (Karyotaki et al., 2017; Petersen et al., 2017), poor adherence may limit their efficacy in the real world. This review highlighted the low levels of treatment adherence in five studies, agreeing with HIC data (e.g., in their review, Andrews et al., 2018 found that iCBT adherence ranged from 6% to 100%). Notably, adherence also tends to be higher in research studies than in real-world scenarios (Baumel et al., 2019). Additionally, DMHI acceptability tends to be lower than that for traditional mental health services (Kaltenthaler et al., 2008). Strategies to improve YP’s engagement could involve co-designing interventions with YP, as highlighted by WHO guidelines (WHO, 2020). Co-design could also be key to ensure user buy-in, and to ensure that digital technologies are contextually and culturally relevant, and are integrated and adopted effectively into health systems (Economist Impact, 2022; NHS Race and Health Observatory, 2023). Effective co-design should utilise a multidisciplinary and multisectoral approach involving ministries of health, clinicians, carers and YP with lived experience of mental health conditions to capture the broad range of stakeholders involved in the digital mental health ecosystem (WHO, 2020; Sanz, 2021).

Given the challenges identified above, there is a need for increased research on this topic. Specifically, more rigorous RCTs with larger sample sizes are needed to increase confidence in the clinical significance and power of results, and permit synthesis of high-quality evidence through meta-analysis. Future studies should have a broader geographic coverage (especially focussing on unrepresented areas such as from Oceania, the Caribbean or Central Asia). The scope of studies should also be increased. Studies should focus on a broader range of mental health interventions apart from iCBT. Future research should also include participants with a wider range of psychopathologies, symptom severity, comorbidities and on psychotropic medication to increase the generalisability of study findings and ability to implement findings in real-world healthcare settings.

The quality of studies could be improved by ensuring that studies report standardised effect sizes and statistical significance to allow for findings to be compared across studies and meaningful conclusions to be made. Studies should aim to reduce self-selection during recruitment, attempt to reduce loss to follow-up, and ensure that participants and researchers are blinded. Studies should also focus on neglected yet important aspects of DMHIs, such as reporting on intervention design to evaluate the impact of design elements on treatment efficacy, and cost-effectiveness to improve potential for implementation. Studies should also report follow-up periods and aim to produce long-term follow-up data by ensuring follow-up for over 6 months. Such efforts could generate new and important findings about methods of action for effective interventions, enhance intervention acceptability, improve intervention generalisability and ensure that new technologies are more sustainable and can be better integrated into existing mental health systems.

It is also key for future studies to examine the implementation processes of intervention studies to help support understanding on their effectiveness and mechanisms of impact. As per UK Medical Research Council guidelines (Craig et al., 2008; Skivington et al., 2021), ensuring that implementation is considered early in the intervention process and throughout intervention development, feasibility testing, process and outcome evaluation are key. This increases the potential of developing interventions that can be adopted and sustained in a real-world context.

### Limitations

This review has a number of limitations. It is notable that four out of the seven included papers were found via handsearching and not identified in the database search. This implies a lack of sensitivity in the search strategy. The author was not able to identify the reason for this, despite ensuring the key terms from hand-searched papers were included in the main search strategy and checking the search strategy with LSHTM library staff. Moreover, due to the large variation in outcome measures, intervention types and study durations, it was not possible to conduct a quantitative synthesis of findings and meta-analysis, which limits the validity of the review’s conclusions. Finally, excluding non-English language studies in the search may have led to the authors missing key articles in other languages.

## Conclusions

The present systematic review is the first to identify and synthesise the current body of literature evaluating the clinical effectiveness of DMHIs for YP in LMICs. The findings suggest the effectiveness of digital technologies, especially iCBT-based interventions, to address depression and anxiety in this population. Importantly, the findings are also consistent with growing evidence on DMHIs from HICs that show potential for DMHIs to improve mental health conditions in YP. However, the evidence in this review is limited to only seven studies and should be treated with caution.

This review, combined with emerging recent evidence, highlights opportunities for DMHIs to address the burden of mental illness and global inequalities in effective mental health care for YP. It also identifies the need to improve the quantity and quality of available evidence on the topic through increased rigorous research. Finally, this review also highlights opportunities to utilise evidence-based policy mechanisms to increase the impact of DMHIs in LMICs.

## Supporting information

Alagarajah et al. supplementary material 1 Alagarajah et al. supplementary material

Alagarajah et al. supplementary material 2 Alagarajah et al. supplementary material

## Data Availability

The author confirms that the data supporting the findings of this study are available within the article and its Supplementary Materials.

## Supplementary material

The supplementary material for this article can be found at http://doi.org/10.1017/gmh.2024.71.
