# Supplementary material for: Digital mental health interventions for treating mental disorders in young people based in low-and middle-income countries: A systematic review of the literature
Source: Glob Ment Health (Camb). 2024 Aug 27;11:e74. doi: 10.1017/gmh.2024.71 (PMC11418075; doi:10.1017/gmh.2024.71)
Supplement: Alagarajah et al. supplementary material 2 — Alagarajah et al. supplementary material [file S2054425124000712sup002.docx]

**Supplementary Material S2**. CASP Quality Assessment Criteria.

|  | CASP criteria (CASP 2020) | | | | | | | | | | | Overall Quality Assessment |
| --- | --- | --- | --- | --- | --- | --- | --- | --- | --- | --- | --- | --- |
| Author | Did the study address a clearly focused research question? | Was the assignment of participants to interventions randomised? | Were all participants who entered the study accounted for at its conclusion? | • Were the participants ‘blind’ to intervention they were given? • Were the investigators ‘blind’ to the intervention they were giving to participants? • Were the people assessing/analysing outcome/s ‘blinded’? | Were the study groups similar at the start of the randomised controlled trial? | Apart from the experimental intervention, did each study group receive the same level of care (that is, were they treated equally)? | Were the effects of intervention reported comprehensively? | Was the precision of the estimate of the intervention or treatment effect reported? | Do the benefits of the experimental intervention outweigh the harms and costs? | Can the results be applied to your local population/in your context? | Would the experimental intervention provide greater value to the people in your care than any of the existing interventions? |  |
| Sun et al. (2022) | Yes - population, intervention, comparator and outcomes described. | Yes (using an a priori randomisation sequence). | No - at 2 months post baseline the intervention group had a 9% drop out rate, whilst the control group had a 18% drop out rate. | Yes - Participants and investigators were blinded to the condition until the allocation was revealed. | No - there were significant demographic differences between the intervention and control groups at baseline. | Yes - identical follow-up period for both groups. | Yes - results reported clearly in a table with P values. Dropout rates reported. However, social support group had a higher dropout rate compared to mhealth group at 2 months post baseline indicating potential for bias. | Yes - 95% confidence intervals reported. | Yes - moderate reduction in anxiety in participants outweighs small amount of students (8.7% of the SS group) reporting mild adverse events. | No - Chinese university students may be culturally different to UK university students (e.g. language or cultural barriers could have affected the extent to which users would access, engage, and benefit from the treatment). Also, contextual factors (Covid pandemic is at a different stage and lockdowns are not occurring in the UK) are different in the UK. However, outcomes are important to UK population. | No - no cost-effectiveness analysis performed. | Moderate |
| Ofoegbu et al. (2020) | Yes - population, intervention, comparator and outcomes described. | Yes - using a sequence generated by computer allocation software. | No - there was a 41% drop out rate. Authors did not specify whether used ITT/per protocol analysis. | No: participants, investigators, people conducting analysis were not blinded to intervention. | Yes - Chi squared test was conducted and showed no statistically significant differences between groups. | Yes - clearly defined study protocol and identical follow-up intervals between groups. | Yes - measured outcomes clearly defined (Beck’s Depression Inventory-II) and using ANOVA to analyse data. | Yes - precision estimates reported with 95% confidence intervals. | Yes - significant treatment effect due to the intervention and no specific harms recorded. | No - Nigerian university students may be culturally different to UK university students (e.g. language or cultural barriers could have affected the extent to which users would access, engage, and benefit from the treatment). However, outcomes are important to UK population. | No - no cost-effectiveness analysis performed. | Moderate |
| Newman et al. (2021) | Yes - population, intervention, comparator and outcomes described. | Yes - block randomisation carried out. However, no detail on whether allocation sequence was hidden from investigators/participants. | Yes - loss to follow-up was reported (41% of treatment participants and 39% of waitlist participants provided posttreatment data). | No - participants/researchers/those conducting analysis were not blinded. | Yes - however no reporting of key factors e.g. age, sex, socio-economic status. | Yes - identical follow-up intervals between intervention and control group. | Yes - power calculation carried out, outcomes were clearly specified, results reported in a table, P values reported. | No - no confidence intervals reported. | Yes - significant treatment effect due to intervention with no specific harms recorded. | No - Indian university students may be culturally different to UK university students (e.g. language or cultural barriers could have affected the extent to which users would access, engage, and benefit from the treatment). However, outcomes are important to UK population. |  |  |
| Moeini et al. (2019) | No - although the authors did specify the population, intervention and outcomes - they did not specify the comparator group. | Yes - authors carried out a simple random sampling method but did not specify if allocation sequenced was concealed. | Yes - loss to follow-up were reported and ITT analysis was used. | No - authors did not report blinding of participants, investigators/assessors. | Yes - there were not statistically significant differences between the baseline characteristics of the groups. | Yes - same follow-up time for both groups. | Yes - results were clearly reported with P values in a table. | No - no confidence intervals reported. | Yes - moderate change in depression scores reported with no reports of adverse events. | No - study only looked at female students, Iranian students may also be culturally different to UK university students (e.g. language or cultural barriers could have affected the extent to which users would access, engage, and benefit from the treatment). However, outcomes are important to UK population. | No - no cost-effectiveness analysis performed. | Moderate |
| Salamanca-Sanabria et al. (2020) | Yes - population, intervention, comparator and outcomes described. | Yes - using a sequence generated by computer allocation software. | Yes - authors describe loss to follow-up and intention to treat analysis was used. | No blinding of participants or researchers described. | Yes - no significant differences in baseline characteristics between groups. | Yes - both groups had identical follow-up times. | Yes - results were clearly reported with P values in a table. | Yes - precision estimates reported with 95% confidence intervals. | Yes - moderate change in depression scores reported with no reports of adverse events. | No - study only looked at female students, Colombian students may also be culturally different to UK students (e.g. language or cultural barriers could have affected the extent to which users would access, engage, and benefit from the treatment). However, outcomes are important to UK population. | No - no cost-effectiveness analysis performed. | Moderate |
| Osborn et al. (2020) | Yes - population, intervention, comparator and outcomes described. | Yes - methods of randomisation were described. | Yes - all participants who entered the study accounted for at its conclusion and intention to treat analysis was used. | Yes - study team was thus blind to the allocation. | No - There were more males in the study skills control (22) than the intervention group (15). | Yes - both groups had identical follow-up times. | Yes - results were clearly reported with P values in a table. | Yes - precision estimates reported with 95% confidence intervals. | Yes - moderate change in scores reported with no reports of adverse events. | No - study only looked at female students, Kenyan students may also be culturally different to UK students (e.g. language or cultural barriers could have affected the extent to which users would access, engage, and benefit from the treatment). However, outcomes are important to UK population. | No - no cost-effectiveness analysis performed. | Moderate |
| Wannachaiyakul et al. (2017) | Yes - population, intervention, comparator and outcomes described. | Yes - methods of randomisation were described. | Yes - all participants who entered the study accounted for at its conclusion. There was no description of whether intention to treat analysis was used. | Yes - study team was blind to the allocation. | N/A - no comparison of baseline characteristics between groups. | Yes - both groups had identical follow-up times. | Yes - results were clearly reported with P values in a table. | No - precision estimates were not reported with 95% confidence intervals. | Yes - moderate change in scores reported with no reports of adverse events. | No - study only looked at mostly male Thai participants in detention. They may be culturally different to UK students (e.g. language or cultural barriers could have affected the extent to which users would access, engage, and benefit from the treatment). In addition, those in detention may be different to the general population (e.g. have more severe depressive symptoms). However, outcomes are important to UK population. | No - no cost-effectiveness analysis performed. | Moderate |
| **Key:**  CASP : Critical Appraisal Skills Programme  RCT : Randomised Control Trial  mHealth:  mobile health  ITT: Intention to Treat  ANOVA: Analysis of variance | | | | | | | | | | | | |
