# Supplementary material for: Digital mental health interventions for treating mental disorders in young people based in low-and middle-income countries: A systematic review of the literature
Source: Glob Ment Health (Camb). 2024 Aug 27;11:e74. doi: 10.1017/gmh.2024.71 (PMC11418075; doi:10.1017/gmh.2024.71)
Supplement: Peer review history

# Editor report: Recommendation: Digital mental health interventions for treating mental disorders in young people based in low-and middle-income countries: A systematic review of the literature — R0/PR2

**DOI:** 10.1017/gmh.2024.71.pr2

**Affiliations:** University of Pittsburgh, United States

Thank you for submitting your manuscript for review. Both reviewers consider the manuscript relevant and well written. However, they have also identified number of concerns in the methodology, results, discussion, and overall structure on the manuscript. Also, the reviewers have provided recommendations on how to improve the manuscript. We invite you to consider the reviewers comments and recommendations and submit a revised manuscript.

# Editor report: Decision: Digital mental health interventions for treating mental disorders in young people based in low-and middle-income countries: A systematic review of the literature — R0/PR3

**DOI:** 10.1017/gmh.2024.71.pr3

**Affiliations:** Johns Hopkins University Bloomberg School of Public Health, United States

No accompanying comment.

# Editor report: Recommendation: Digital mental health interventions for treating mental disorders in young people based in low-and middle-income countries: A systematic review of the literature — R1/PR5

**DOI:** 10.1017/gmh.2024.71.pr5

**Affiliations:** University of Pittsburgh, United States

Thank you for revising the manuscript and responding to the reviewers recommendations.

All the reviewers are satisfied with the revisions and have recommended that we accept the manuscript. 

We are happy to accept the manuscript in its present form and look forward to working with you through the publication process.

# Editor report: Decision: Digital mental health interventions for treating mental disorders in young people based in low-and middle-income countries: A systematic review of the literature — R1/PR6

**DOI:** 10.1017/gmh.2024.71.pr6

**Affiliations:** Johns Hopkins University Bloomberg School of Public Health, United States

No accompanying comment.
